# Flow Cytometry-based Method for Efficient Sorting of Senescent Cells

**DOI:** 10.21769/BioProtoc.4612

**Published:** 2023-04-05

**Authors:** Erwan Goy, Nathalie Martin, Claire Drullion, Laure Saas, Olivier Molendi-Coste, Laurent Pineau, David Dombrowicz, Emeric Deruy, Hélène Bauderlique-Leroy, Olivier Samyn, Joe Nassour, Yvan de Launoit, Corinne Abbadie

**Affiliations:** 1 Univ. Lille, CNRS, Inserm, CHU Lille, Institut Pasteur de Lille, UMR9020-U1277 – CANTHER – Cancer Heterogeneity, Plasticity and Resistance to Therapies, F-59000 Lille, France; 2 Univ. Lille, Inserm, CHU Lille, Institut Pasteur de Lille, U1011- EGID, F-59000 Lille, France; 3 Univ. Lille, CNRS, Inserm, CHU Lille, Institut Pasteur de Lille, US41 – UAR 2014 – PLBS, F-59000 Lille, France

**Keywords:** Senescence, Flow cytometry, Sorting, FSC, SSC, SA-β-Gal, C_12_FDG, Nozzle

## Abstract

Cellular senescence is a reprogrammed cell state triggered as an adaptative response to a variety of stresses, most often those affecting the genome integrity. Senescent cells accumulate in most tissues with age and contribute to the development of several pathologies. Studying molecular pathways involved in senescence induction and maintenance, or in senescence escape, can be hindered by the heterogeneity of senescent cell populations. Here, we describe a flow cytometry strategy for sorting senescent cells according to three senescence canonical markers whose thresholds can be independently adapted to be more or less stringent: (i) the senescence-associated-β-galactosidase (SA-β-Gal) activity, detected using 5-dodecanoylaminofluorescein Di-β-D-galactopyranoside (C_12_FDG), a fluorigenic substrate of β-galactosidase; (ii) cell size, proportional to the forward scatter value, since increased size is one of the major changes observed in senescent cells; and (iii) cell granularity, proportional to the side scatter value, which reflects the accumulation of aggregates, lysosomes, and altered mitochondria in senescent cells. We applied this protocol to the sorting of normal human fibroblasts at the replicative senescence plateau. We highlighted the challenge of sorting these senescent cells because of their large sizes, and established that it requires using sorters equipped with a nozzle of an unusually large diameter: at least 200 µm. We present evidence of the sorting efficiency and sorted cell viability, as well as of the senescent nature of the sorted cells, confirmed by the detection of other senescence markers, including the expression of the CKI p21 and the presence of 53BP1 DNA damage foci. Our protocol makes it possible, for the first time, to sort senescent cells from contaminating proliferating cells and, at the same time, to sort subpopulations of senescent cells featuring senescent markers to different extents.

Graphical abstract

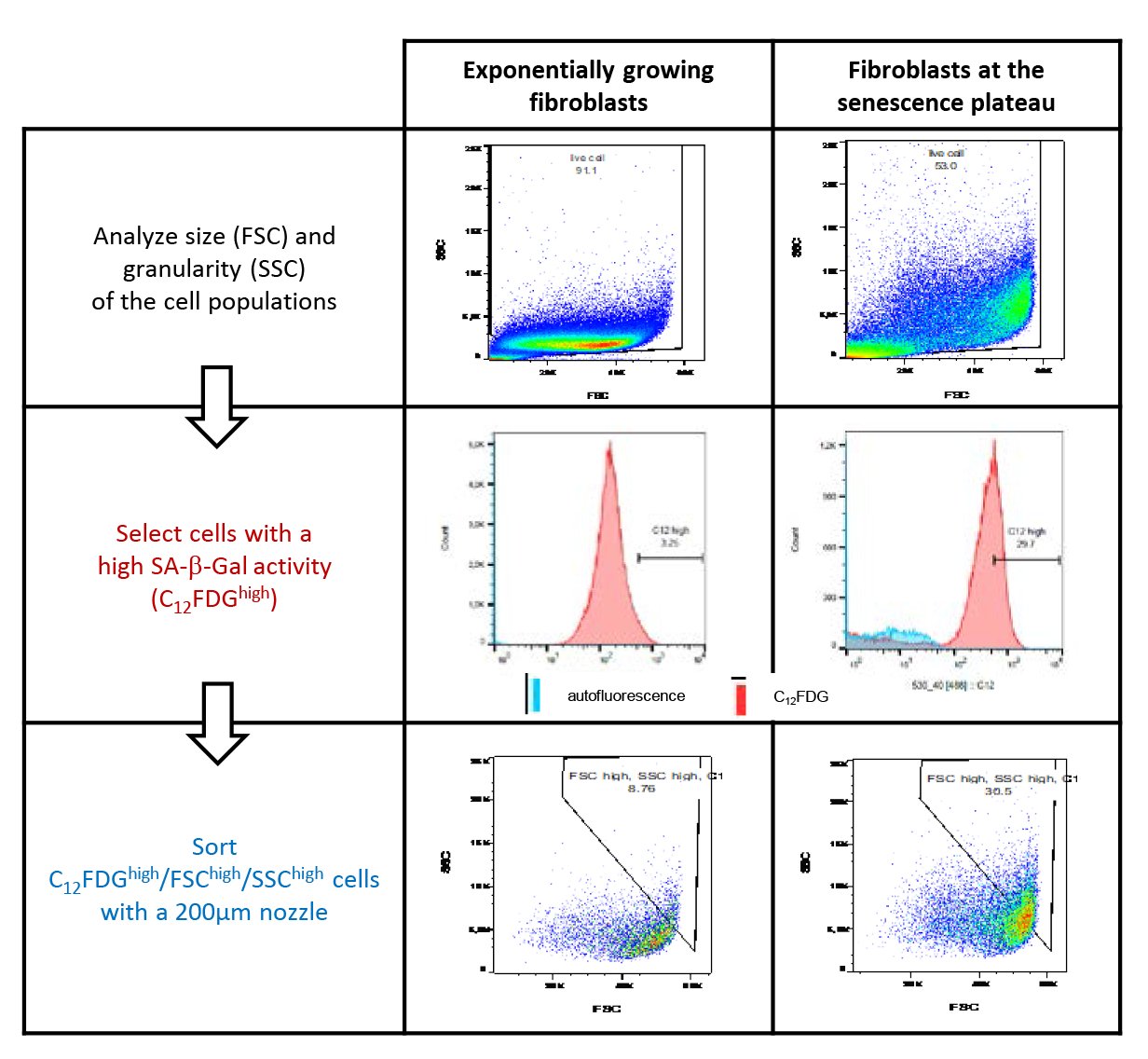

## Background

Cellular senescence can be defined as a reprogrammed cell state triggered to adapt to a variety of stresses, most often those affecting the genome integrity. The term *cellular senescence* thus encompasses i) replicative senescence, which is associated with shortened and dysfunctional telomeres, ii) stress-induced premature senescence (SIPS) in response to oxidative stress, iii) oncogene-induced senescence (OIS) in response to the activation of some oncogenes (mostly components of the RAS pathway), and iv) therapy-induced senescence (TIS) triggered in response to several anticancer chemotherapeutics or to radiotherapy. There is ample evidence that senescent cells accumulate in most tissues with age and also at sites of many pathologies, and that they contribute to the development of several age-associated dysfunctions and pathologies (for reviews see Abbadie et al., 2017; Di Micco et al., 2021; Tripathi et al., 2021).

 Senescent cells undergo multiple molecular and phenotypic changes based on both epigenetic and genetic reprogramming. They become larger and more spread than their proliferating counterparts. They also stop dividing, most often arrested at the G1 phase of the cell cycle. Their secretomes are quantitatively and qualitatively modified, with an enrichment in pro-inflammatory cytokines and chemokines, growth factors, and matrix-remodeling enzymes. They suffer from chronic oxidative stress and accumulate oxidized cell components, such as lipofuscin. They are also subject to chronic endoplasmic reticulum stress, which activates the unfolded protein response pathway. Moreover, they accumulate unrepaired DNA damage, constantly activating some signaling and repair pathways, such as the DNA damage response and/or the single-strand break response pathways, leading to the activation of the p21 and/or p16 cyclin-dependent kinase inhibitors (encoded by *CDKN1A* and *CDKN2A*, respectively). Some leakage of nuclear chromatin in the cytosol may occur, leading to the activation of the cGAS-STING pathway. Under such stress conditions, senescent cells show an increased autophagic activity reflected by an increased mass of phagosomes, phagolysosomes, and lysosomes, and an increased activity of lysosomal enzymes, such as β-galactosidase (for reviews see Malaquin et al., 2016; Abbadie et al., 2017; Hernandez-Segura et al., 2018; Li and Chen, 2018; Abbadie and Pluquet, 2020; Young et al., 2021). Several of these molecular and phenotypic changes are used as markers to identify senescent cells in mixed cell populations using in vitro culture assays, as well as in tissue sections. The most common among these markers are the following: the senescence-associated-β-galactosidase (SA-β-Gal) activity, with reflects the lysosomal mass; the p16 expression, which is a marker of the cell cycle arrest; and the expression of some cytokines, such as IL-6, which reflect the secretome change. However, since none of these molecular and phenotypic alterations are strictly specific to cellular senescence, experts agree that more than one marker must be recorded to assert that a cell is indeed senescent (Hernandez-Segura et al., 2018).

 Although considerable progress has been made in the past 20 years, not all of the molecular pathways contributing to senescence induction, maintenance, and escape have been entirely elucidated. To fill this knowledge gap, assays on in vitro cultures of primary cells or cell lines induced into senescence have been commonly used. While this is still relevant, their use has some limitations. According to our experience, a population of senescent cells is never homogenous. It can comprise senescent cells featuring senescent markers to different extents. To make matters worse, senescent cells can be mixed either with still proliferating pre-senescent cells or with post-senescent ones, which reproliferate after senescence escape. Facing these limitations, we have set up a protocol for sorting senescent cells by flow cytometry.

 In our previous studies, we sorted different subpopulations of senescent normal human epidermal keratinocytes (NHEKs) in order to get insights on their outcomes, i.e., stability, cell death, or neoplastic escape. To this end, we sorted the cells according to their forward and side scatter (FSC and SSC) factors, which reflect cell size and granularity, respectively. We observed a global increase in these two parameters in a population of NHEKs at the senescence plateau compared with an early passage of proliferating NHEKs. However, we found a continuous spectrum of FSC and SSC values within the population at the senescence plateau, evidencing a great variability in the extent of the senescent phenotype. We then sorted different subpopulations according to their FSC and SSC values. We showed that senescent cells with the highest FSC and SSC values were most likely to undergo cell death by autophagy, whereas those with values just below the highest were all alive and had a higher potential to escape senescence and give rise to pre-transformed cells (Deruy et al., 2010, 2014; Gosselin et al., 2009a, 2009b; Nassour et al., 2016).

 Very recently (Goy et al., 2022), we have improved our sorting protocol by adding a third parameter to better discriminate senescent cells: the SA-β-Gal activity. This enzymatic activity can be detected using C_12_FDG, as initially described by Debacq-Chainiaux et al. (2009). C_12_FDG is a cell-permeant fluorogenic substrate of β-galactosidase. It is composed of a di-β-D-galactopyranoside (FDG) and a 12-carbon lipophilic moiety (C_12_). The β-Gal-mediated hydrolysis of FDG generates the fluorescent signal. According to the manufacturer, the C_12_ moiety helps to retain the fluorescent product in the cells, probably by insertion into cellular membranes. It is noteworthy that we applied this technique to normal human fibroblasts. These cells are the most commonly used model in the senescence field, but they pose an additional problem regarding flow cytometry sorting: their very large sizes at senescence, larger than those of senescent NHEKs. Because of this, we did not succeed in sorting senescent fibroblasts on cytometers equipped with a standard nozzle of 100–130 µm in diameter. Herein, we present evidence that normal human dermal fibroblasts (NHDFs) at replicative senescence can be efficiently sorted using a sorter equipped with a 200 µm nozzle, based on three senescence markers: size, granularity, and SA-β-Gal activity. Our technique is sufficiently sensitive to separate senescent sub-populations of different sizes, granularity, and/or SA-β-Gal activity, which may have different properties, behaviors, and/or outcomes. It could also be used for a pseudo-time reconstruction, to discriminate and/or sort cells that are in deep senescence from those having just acquired the senescent phenotype.

## Materials and Reagents

1. Pre-sorting tubes and post-sorting collecting tubes: polypropylene round-bottom tubes with snap caps (BD, catalog number: BD352063)
2. Polypropylene centrifuge tubes (Greiner, catalog number: 188271)
3. Cover slides
4. 5, 10, and 25 mL pipettes
5. Micropipettes with tips
6. T75 cell culture flasks (Falcon, catalog number: 353136)
7. Normal human dermal fibroblasts (NHDFs) (Promocell, catalog number: C-12300)
8. Fibroblast growth medium-2 BulletKit^TM^ (Lonza, catalog number: cc-3132)
9. Trypsin/EDTA (TE) (Gibco, catalog number: R001-100)
10. Trypan blue (Gibco, catalog number: 15250-061)
11. Trypsin neutralizer solution (TN) (Gibco, catalog number: R002-100)
12. 5-dodecanoylaminofluorescein di-β-D-galactopyranoside (C_12_FDG) (Invitrogen, catalog number: D2893); prepare a C_12_FDG 20 mM stock solution in DMSO
13. Pre-sort buffer (BD, catalog number: BD 563503); the pre-sort buffer must be cold: put it at 4 °C one day before the experiment
14. Fetal bovine serum (FBS) (Eurobio, catalog number: CVFSVF00-0U), heat-inactivated at 56 °C for 30 min
15. Sterile DPBS (no calcium, no magnesium) (Gibco, catalog number: 14190-144)

## Equipment

1. Refrigerated centrifuge (Eppendorf, centrifuge 5810R or equivalent)
2. CO_2_ incubator (Thermo Scientific, HeraCell 150i, or equivalent)
3. Class II biosafety cabinet (Thermo Scientific, HeraSafe KS12, or equivalent)
4. Inverted phase contrast microscope (Zeiss, Axio Vert.A1, or equivalent)
5. Cell sorter: INFLUX V7 sorter (Becton Dickinson, ref: 646500M8 – 3B3R5V5YG SD AE) equipped with a 200 µm nozzle or equivalent. If your objective is to culture the sorted cells, wash the cell sorter before sorting, carefully applying all the steps recommended by the cytometer’s manufacturer.
  Sorting senescent cells is challenging due to their large sizes, and even more so when using fibroblasts, which are large compared with other cell types. Therefore, sorters equipped with a standard 100–130 µm nozzle are inadequate. Indeed, we have measured the diameter of suspended NHDFs at the replicative senescence plateau (Figure 1A) and in SIPS (Figure 1B). The mean diameter of cells in both senescence states was approximately 50 µm, representing approximately a two-fold increase compared with exponentially growing cells, with some senescent cells reaching a diameter of up to 100 µm (Figure 1C–F). Usually, it is recommended by sorter manufacturers that the size of the nozzle be five-fold that of the cell diameter, to avoid generating a hydrodynamic rebound when the cell goes through the nozzle. This would induce parasite vibrations of the flux leading to the instability of the breakoff point and, consequently, to spreading of drop deflection. However, practice shows that the nozzle diameter can usually be reduced to two-fold the diameter of cells before drop deflection spreading is observed. Noteworthy, very few sorters are provided with or even designed to run with nozzles larger than 130 µm in diameter. The INFLUX v7 cell sorter (Becton Dickinson) we chose to use is designed to run with a 200 µm nozzle, which was efficient in providing stable deflection of senescent fibroblasts in our hands.
  The cell sorter is equipped with a supplemental circulating water–cooling device supplied with the instrument (Thermo Scientific HAAKE A10 – 152510101)
  **Figure 1.**
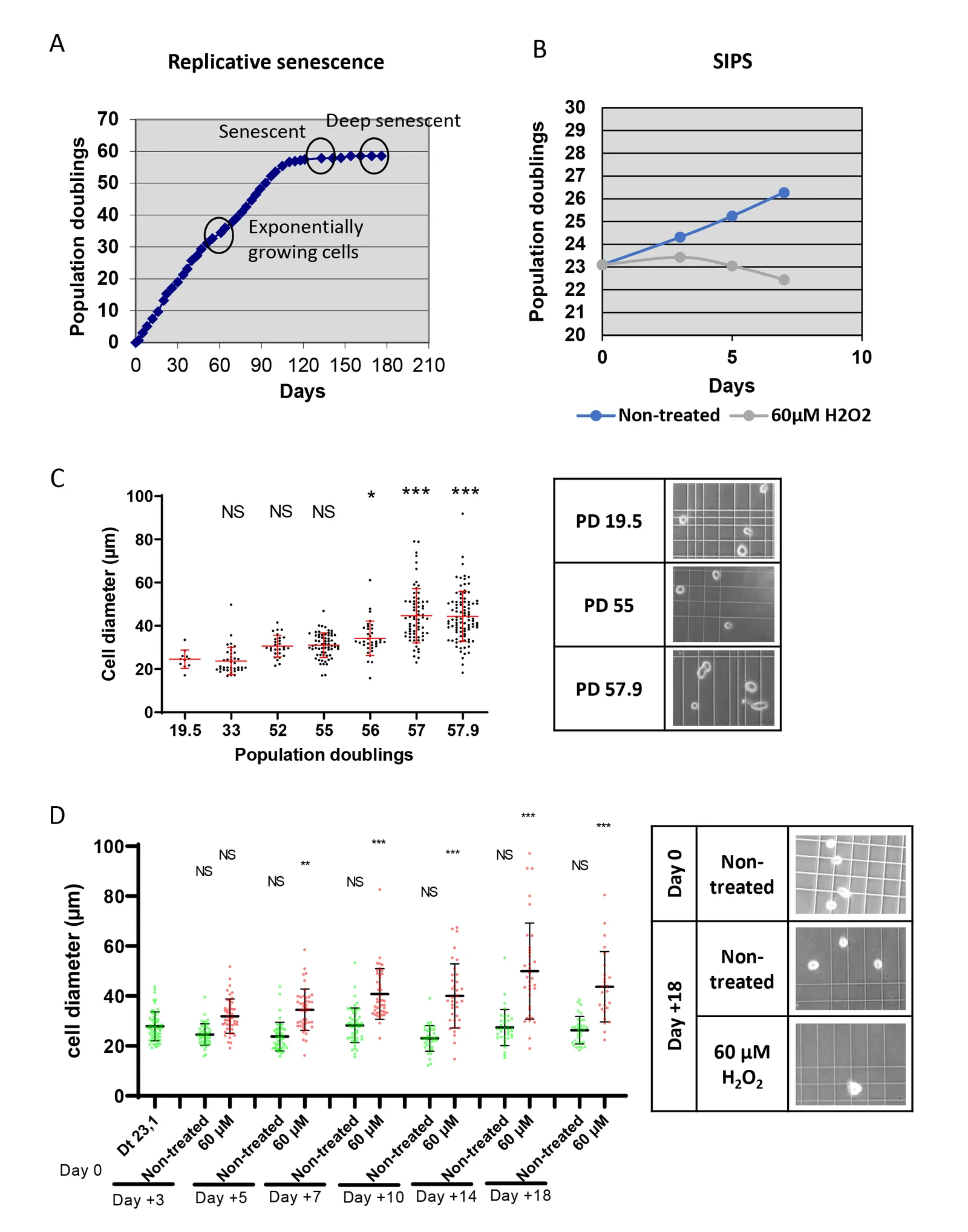
Characteristics of senescent normal human dermal fibroblasts (NHDFs). **A.** Growth curve of NHDFs up to the replicative senescence plateau. The time at which the cells were collected for sorting is indicated. **B.** Growth curves of control NHDFs and NHDFs induced into stress-induced premature senescence (SIPS) by a 60 µM H_2_O_2_ daily treatment. **C–D.** Cell diameters of NHDFs in suspension by their population doubling number (C) or by the H_2_O_2_ treatment duration (the day after the beginning of the treatment; D). Each dot represents the diameter of one cell. The bars represent the mean ± SD. A Kruskal-Wallis analysis was done. NS: non-significant; **: p < 0.01; ***: p < 0.0001. Right panels: representative phase contrast microscopy images used to measure cell diameters.
6. Malassez chambers (Marienfeld, catalog number: 0640610, or equivalent); if you use a cell counter device, check if it can count large cells

## Software

1. BD FACS Software Ink (developed and supplied by Becton Dickinson^®^)
2. FlowJo V10.8.1 (developed by FlowJo LLC and supplied by Becton Dickinson^®^)

## Procedure

The protocol described below is for sorting out fully senescent NHDFs from among NHDFs at the replicative senescence plateau. These cells are sorted as C_12_FDG^high^/FSC^high^/SSC^high^. We also used the same protocol to sort NHDFs induced into senescence by X-ray irradiation (Goy et al., 2022).

- **Cell culture**
  To be able to objectively delineate the sorting gates corresponding to senescent cells, it is mandatory to use a negative control and advisable to have a positive one. The best negative control is to use cells from the same cell culture at the earliest possible passage. There is no obvious positive control for replicative senescence. However, cells at replicative senescence can be used as a positive control for SIPS, OIS, or TIS. Of course, classical cytometry controls must also be included (i.e., unstained cells in our protocol) to take into consideration the level of autofluorescence. Note that this autofluorescence level can be higher in senescent cells than in proliferating ones because of autofluorescent oxidized component aggregates, like lipofuscin, that accumulate in senescent cells (Evangelou et al., 2017).
  1. NHDFs are primary cells. Cultivate them at 37 °C in an atmosphere of 5% CO_2_ and at atmospheric O_2_ pressure, in fibroblast growth medium-2 (160 µL/cm^2^). Always proceed to subculturing before the cells reach 70% confluency. For this, rinse them with TE preheated at 37 °C, add fresh TE (40 µL/cm^2^), and incubate at 37 °C for 5 min in case of exponentially growing cells or 10 min for cells having reached the senescence plateau. Then, add the same volume of TN preheated at 37 °C. Collect the cells and pellet them by centrifugation at 90 *× g* for 5 min at room temperature. Replate at 3,500 cells/cm^2^ in case of exponentially growing cells, and at 2,000 cells/cm^2^ for cells at replicative senescence. Under these conditions, exponentially growing NHDFs will reach the replicative senescence growth plateau after 50–70 population doublings, i.e., in approximately five months (Figure 1A). At this plateau, senescent cells can be easily recognized by their increased sizes observable under a phase contrast microscope.
  2. Freeze early passage cells and put them again in culture a few days before the sorting to use them as a negative control.
- **C_12_FDG staining**
  The incubation time and concentration of C_12_FDG should be adapted to the cell type. For NHDFs, we incubate the cells with 33 µM C_12_FDG at 37 °C for 2 h. For NHEKs, we use a 16 µM C_12_FDG concentration and incubate the cells at 37 °C for 16 h. Mind that high concentrations of C_12_FDG or long incubation times may be toxic, depending on the cell type. The efficacy and non-toxicity of the C_12_FDG staining protocol can be checked under an inverted epifluorescence microscope.
  1. Dilute C_12_FDG from the stock solution in the culture medium preheated at 37 °C to obtain a 33 µM concentration. For unstained controls, dilute the same volume of DMSO (the C_12_FDG vehicle) in the culture medium preheated at 37 °C and put it on the cells at 100 µL/cm^2^.
  2. Incubate for 2 h at 37 °C.
- **Cell harvesting**
  All the steps must be performed in the dark to avoid photobleaching of C_12_FDG.
  1. Remove the C_12_FDG- or DMSO-containing culture medium. Rinse the cells twice with 30 µL/cm^2^ of sterile DPBS at room temperature.
  2. Remove the DPBS and harvest the cells by incubating them at 37 °C with TE preheated at 37 °C (40 µL/cm^2^) until the cells are detached and separated from each other (check under an inverted phase contrast microscope). Note that the incubation time may be increased by approximately 50% for senescent cells compared with that for proliferating cells.
  3. Neutralize TE by adding TN preheated at 37 °C (a volume of TN for a volume of TE).
  4. Count the cells (to be able to resuspend them at the desired concentration afterwards).
  5. Centrifuge the cells at 90 *× g* for 5 min at 4 °C.
  6. Rinse the pellets with 500 µL of cold pre-sort buffer.
  7. Centrifuge the cells at 90 *× g* for 5 min at 4 °C.
  8. Resuspend the cells in cold pre-sort buffer to the concentration of 4 × 10^6^ cells/mL.
  9. Keep the tubes on ice until sorting to protect the fluorescent SA-β-Gal product from degradation.
- **Cell sorting**
  We propose to sort senescent cells using the following three parameters: size, granularity, and SA-β-Gal activity. Four gates will have to be designed (Figure 2). The first one is used, as usually in flow cytometry sorting, to separate live cells from the debris (Figure 2A), the second to exclude doublets (Figure 2B), and the third to define the C_12_FDG^high^ cells. This gate is delineated by comparing the dot-plot of C_12_FDG-stained senescent cells to those of both unstained senescent cells and C_12_FDG-stained non-senescent cells (Figure 2C). The fourth gate serves to delineate the largest and most granular cells within the C_12_FDG^high^ population, using their FSC and SSC values (Figure 2D). The stringency of the two last gates can be adapted, depending on your objectives. Moreover, these two last gates can be subdivided to sort different senescent subpopulations featuring the three senescence markers to different extents. In that case, we recommend delineating the gates such that they are not strictly adjacent, in order to obtain sufficiently different subpopulations.
  Proceed to cell sorting as soon as possible after cell harvesting. Use a BD INFLUX V7 or an equivalent cell sorter, equipped with a 200 µm nozzle. Sorting parameters must be tuned adequately to the nozzle size, according to the cytometer’s manufacturer recommendations. Pre-sorting tubes and post-sorting collecting tubes must be in polypropylene and coated with heat-inactivated FBS to avoid cell adhesion to the tube walls. Moreover, we recommend regularly shaking the pre-sorting tubes gently during the sorting process to resuspend the cells that could have sedimented.
  **Figure 2.**
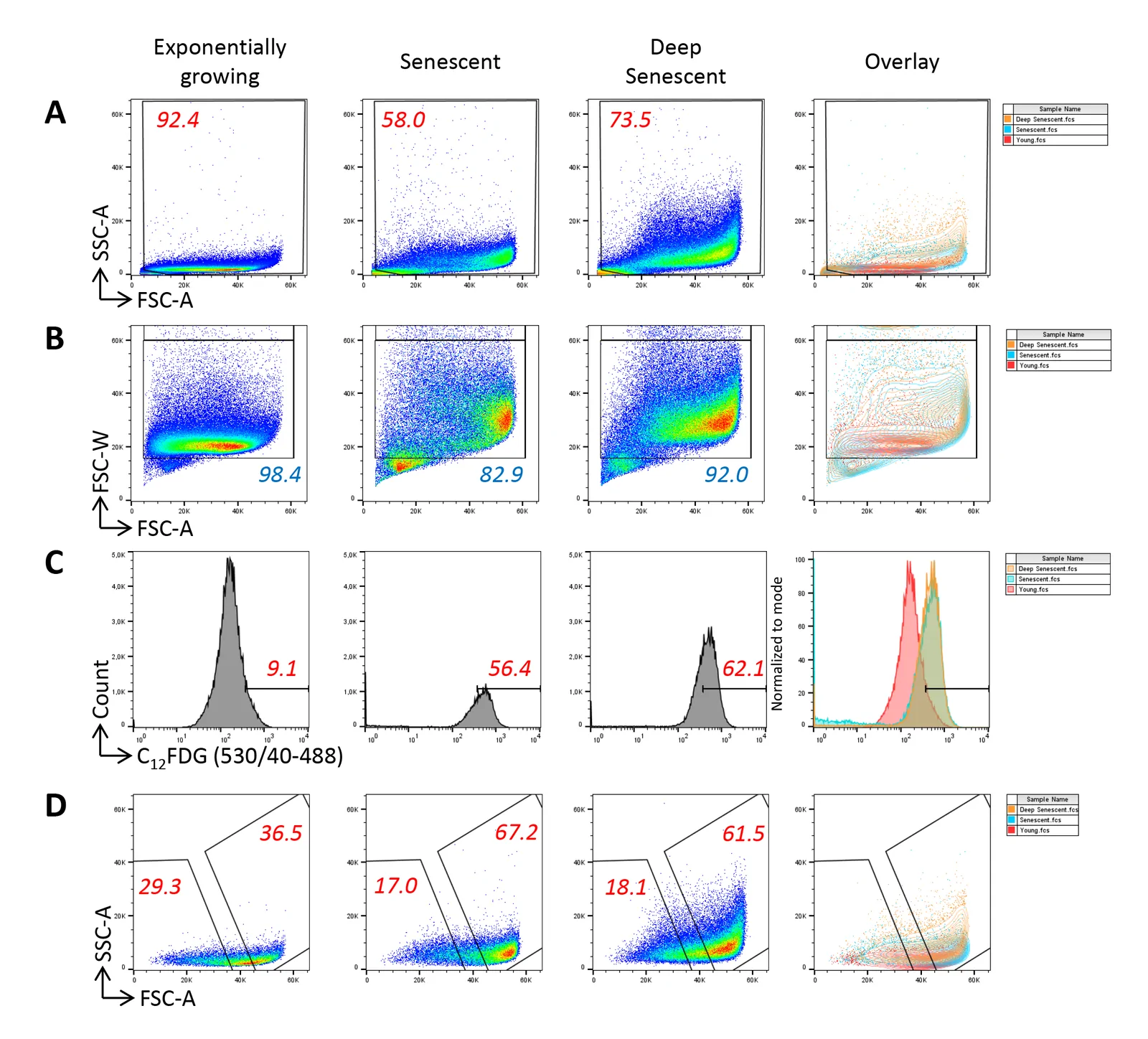
Gating strategy for the sorting of senescent normal human dermal fibroblasts at the replicative senescence plateau. **A.** The first gate is set to eliminate debris using the forward scatter (FSC) and side scatter (SSC) values. Note that large and granular cells are much more numerous in the population at the replicative senescence plateau than in the population of exponentially growing cells. **B.** The second gate serves to exclude doublets using the forward scatter–width (FSC-W) and forward scatter–area (FSC-A) values. **C.** The third gate is set to define the C_12_FDG^high^ population. It is delineated by considering the autofluorescence level of unstained cells and the fluorescence level of C_12_FDG-stained exponentially growing cells. **D.** The fourth gate is used to delineate the FSC^high^/SSC^high^ cells within the C_12_FDG^high^ population that will be sorted.
  1. Prepare the pre-sorting polypropylene tubes and post-sorting polypropylene collecting tubes: fill the tubes with FBS previously inactivated by a 30 min incubation at 56 °C. Let the tubes be coated during 30 min at 37 °C.
  2. Prepare the cell sorter for acquisition of FSC, SSC, and C_12_FDG fluorescence [the 530/40 filter–5/6-fluorescein isothiocyanate (FITC) equivalent, 488 nm laser], according to the manufacturer’s instructions.
  3. Cool the pre- and post-sorting tubes to 4 °C using the circulating water–cooling device.
  4. Analyze the FSC vs. SSC parameters of the unstained batches of NHDFs at early passages and those at the senescence plateau. Optimize the amplification parameters and scales to visualize the cells of all FSC and SSC values. Given very large sizes of senescent cells, instrument signal processing is particularly challenged and the linearity of the scale for FSC may be questionable. However, in contrast to the classical digital display on most cell sorters, the INFLUX V7 cell sorter displays analogic signals, theoretically ensuring that the linearity of the scales is preserved despite extreme parameters adjustments. Delineate the first gate to exclude debris (Figure 2A).
  5. Analyze the FSC-W vs. FSC-A parameters to delineate a singlet cell gate. The heterogeneity of cell sizes requires particular attention to delineate doublets’ exclusion on FSC-W/FSC-A parameters, with an unusual necessity to draw a large gate not to exclude the cells with high FSC-W values (Figure 2B). Indeed, these cells may be doublets, but also singlet large cells, i.e., senescent cells. In order to determine whether the cells with the highest FSC-W values were doublets or singlet large cells, we analyzed their C_12_FDG, FSC, and SSC values in comparison with those of cells with the lowest FSC-W values. Regarding exponentially growing cells, we found that those with the highest FSC-W values had a higher C_12_FDG signal than the cells with the lowest FSC-W values. A majority of these cells had low FSC and SSC values, indicating that these were indeed mainly doublets (Figure 3). Regarding cells at the senescence and deep senescence plateau, the cells with the highest FSC-W values had the same C_12_FDG signal than those with lower FSC-W values. Compared with exponentially growing cells, a higher proportion of them had high FSC and SSC values, suggesting that a portion of them were doublets of still small cells and the others were large singlet cells, i.e., senescent cells (Figure 3).
    **Figure 3.**
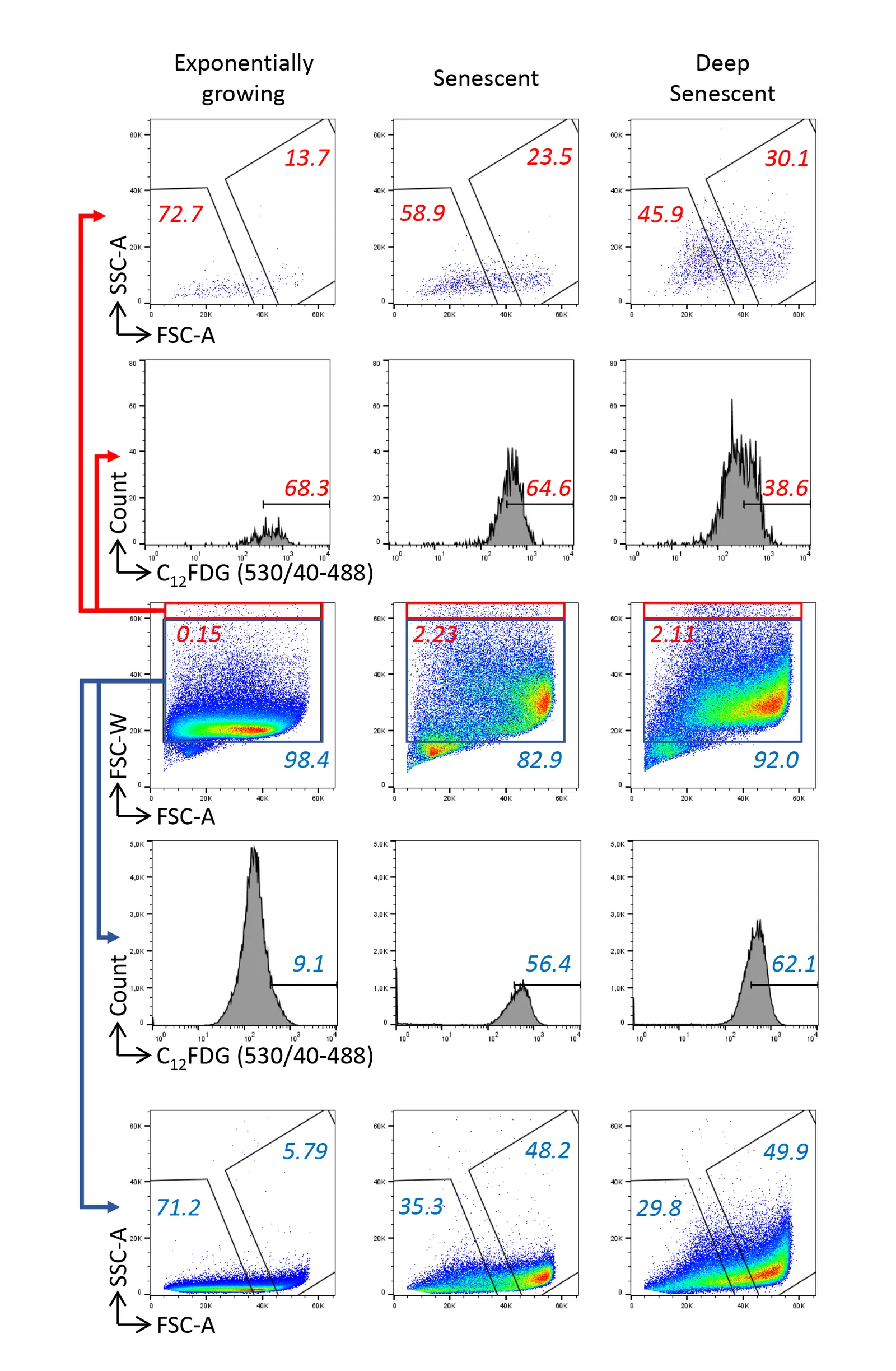
Checking the accuracy of the doublet exclusion strategy. Cells were analyzed by their forward scatter–width (FSC-W) and forward scatter–area (FSC-A) values. Gates of assumed *singlets* (blue) and *doublets* (red) were delineated. Cells in these gates were further analyzed for their C_12_FDG, forward scatter (FSC), and side scatter (SSC) values.
  6. Analyze the green fluorescence of all stained and unstained batches in a Log scale histogram. Delineate the third gate of C_12_FDG^high^ cells by comparing the fluorescence intensity of C_12_FDG-stained senescent cells to that of (i) unstained senescent cells (to evaluate autofluorescence) and (ii) C_12_FDG-stained early passage cells (Figure 2C).
  7. Then, analyze the FSC vs. SSC parameters of the C_12_FDG^high^ cells only. Delineate the gate of FSC^high^/SSC^high^ cells (Figure 2D).
  8. Sort the C_12_FDG^high^/FSC^high^/SSC^high^ cells using the parameters indicated in Table 1, at 500–1,000 events/s (it is possible to go up to 2,000 events/s, but a lower sorting speed is better to avoid the deflection stream spreading). Begin by checking (using the unstained cells at the senescence plateau) that the deflection stream is stable and that cells are properly deflected to the collecting tube. To do that, cover the collecting tube with a cover slide and check that the drop falls at its center.
    **Table 1.** Sorting parameters

Nozzle diameter	Sheath pressure	Sort device	Piezo amplitude	Drop delay	Sort mode	Drop envelope	Sort objective	Phase mask	Extra coincidence bits	Drop frequency (kHz)
200 µm	3.7 psi	2 tube holder—2-way sort	4.01	15.6711	1.0 Drop Pure	1.0 Drop	Purify	16/16	4	6.30
  9. Collect the cells at 4 °C in the circulating water–cooling device and proceed to any further treatment (e.g., protein extraction or plating for follow-up).

## Data analysis

Here, we sorted a bit more than 60% of the cells with the highest FSC and SSC values amongst approximately 60% of the cells with the highest C_12_FDG signal in a population of NHDFs at the replicative senescence plateau, either at the beginning of the plateau or several weeks after the plateau had been established (which is called deep senescence). These gates can be adapted to be more stringent. For example, in an experiment shown in the graphical abstract, we sorted only 30% of the cells with the highest FCS and SSC values within 30% of the cells with the highest C_12_FDG signal.

Since the sorting of large cells can be challenging, we checked the sorting efficiency of our protocol. The sorting efficiency provided in the sort report (Table 2) was very high, between 86.5% and 91.3%. To validate this sorting efficiency, we manually counted the number of cells in the collecting tubes (using a Malassez chamber) and calculated the percentage of cells that were actually sorted (Table 3). Our results were very similar to those provided by the cell sorter. To challenge the sorter’s ability to sort rare senescent cells, we mixed 50% of exponentially growing cells with 50% of cells at the senescence plateau or at the deep senescence plateau. We found that the sorting efficiencies were similar (Tables 2 and 3).

**Table 2.** Sort reportsTable 3. Sorting efficiency

	Number of cells in the gate	Number of cells in the collecting tube	Sorting efficiency (%)
Cells at the senescence plateau	21,852	20,000	91.52
Cells at the deep senescence plateau	33,859	32,000	94.51
Mix (50/50) of exponentially growing cells and cells at the senescence plateau	6,936	6,000	86.51
Mix (50/50) of exponentially growing cells and cells at the deep senescence plateau	9,403	8,000	85.08
		Mean	89.40
		SD	4.38

**Figure 4.**
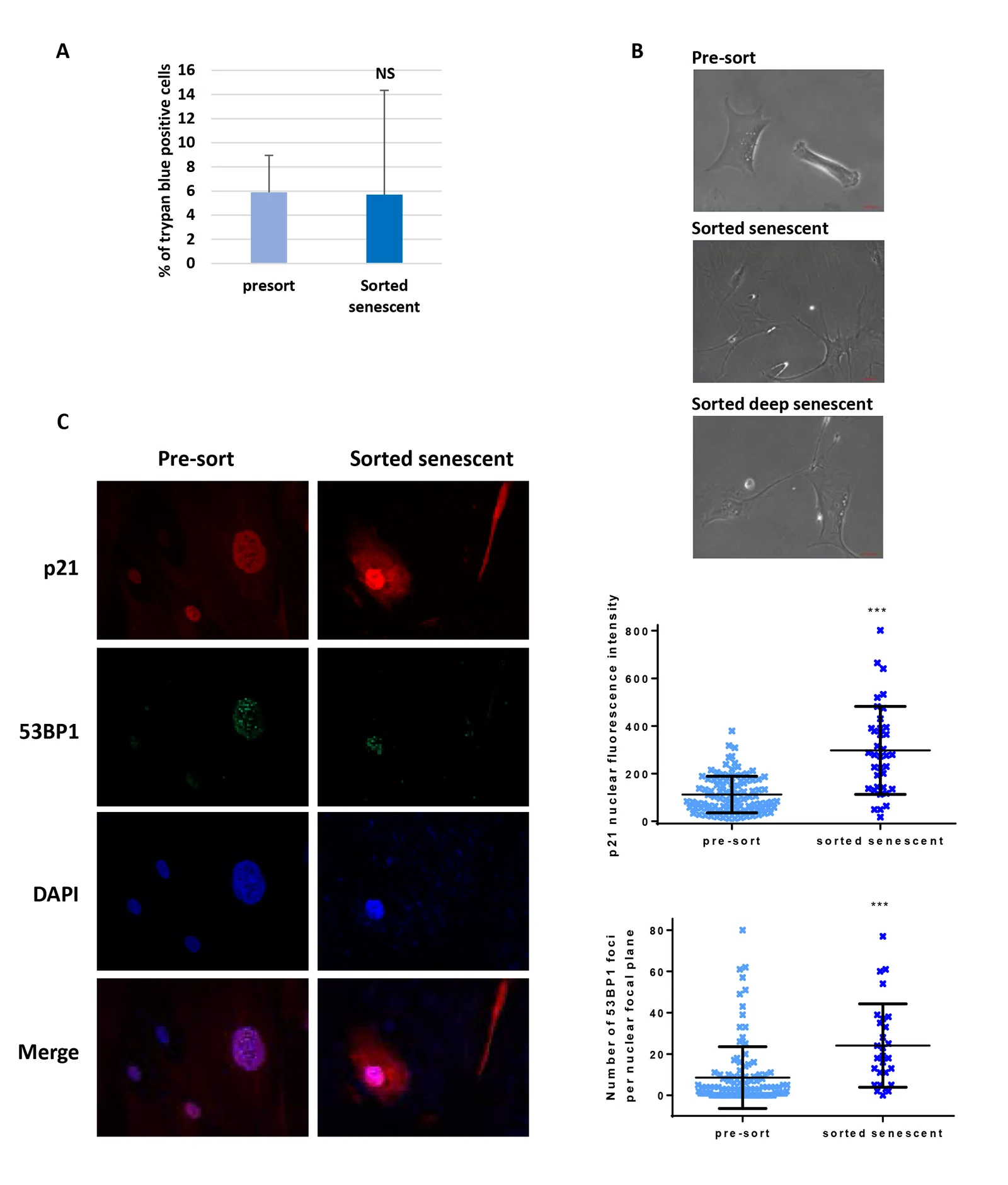
Characteristics of sorted senescent normal human dermal fibroblasts. **A.** Cell death levels were determined by manually counting trypan blue (15250-061, Gibco) positive cells amongst a mean of 57 cells from pre-sort cell populations compared to a mean of 15 cells from sorted cells, in two independent sorts, one with cells at the beginning of the senescence plateau, the other with cells in deep senescence. The bars represent the means ± SD of these counts. A t-test was performed to compare the sorted populations to the pre-sort ones. NS: non-significant difference. **B.** Phase contrast images of pre-sorted cells or cells plated after sorting. Scale bars: 50 µm. **C.** Immunostaining of p21 and 53BP1 in the sorted population compared with the pre-sorted population. Left: representative immunostaining images. Upper right: quantification of the p21 nuclear staining intensity. Each dot represents the nuclear fluorescence intensity of one cell. The bars represent the means ± SD of at least 28 cells. A Mann-Whitney test was done to compare the two populations. *** indicates p < 0.001.

Another delicate issue could have been the viability of the sorted senescent cells. To address this question, we assessed the viability of the sorted cells compared with the pre-sorted total population using trypan blue staining. We observed an about 2-fold increase in the percentage of trypan-blue-positive cells in the sorted population compared to the pre-sorted one, however this difference was not statistically significant, and the level of cells with an altered membrane permeability revealed by the trypan-blue staining remained low, at about 15% (Figure 4A). Moreover, sorted senescent cells, and even deep senescent ones, were able to replate (Figure 4B).

Finally, we checked that the sorted cells were indeed senescent. We first examined their morphology after plating using phase contrast microscopy. Sorted cells were very large and spread, with refringent vesicles in the cytoplasm and often big nucleoli (Figure 4B). Second, we searched for the expression of p21, the main cyclin-dependent kinase inhibitor involved in the cell cycle arrest of replicative senescent cells, using immunofluorescence. We found an increase of approximately 2.6-fold in the nuclear expression of p21 in sorted cells compared with pre-sorted cells at the senescence plateau (Figure 4C). Finally, we investigated the telomere status of the sorted cells by enumerating 53BP1 foci, which mainly form at the shortened telomeres. The number of 53BP1 foci per nucleus increased by approximately 2.8-fold in sorted cells compared with pre-sorted cells at the senescence plateau (Figure 4C).

Altogether, these results demonstrate that our sorting strategy makes it possible to efficiently sort senescent fibroblasts, which remain viable and able to replate, and whose senescent nature is confirmed by the presence of molecular senescent canonical markers. The protocol we developed enables the sorting of senescent cells using three parameters. We presume that it is possible to include additional markers, provided that they can be detected using a vital fluorochrome.

## References

[1] Abbadie C. and Pluquet O. (2020). Unfolded Protein Response(UPR) Controls Major Senescence Hallmarks. Trends Biochem Sci 45(5): 371-374. 10.1016/j.tibs.2020.02.005 32311331

[2] Abbadie C., Pluquet O. and Pourtier A. (2017). Epithelial cell senescence: an adaptive response to pre-carcinogenic stresses? Cell Mol Life Sci 74(24): 4471-4509. 10.1007/s00018-017-2587-9 PMC11107641 28707011

[3] Debacq-Chainiaux F., Erusalimsky J. D., Campisi J. and Toussaint O. (2009). Protocols to detect senescence-associated beta-galactosidase(SA-betagal) activity, a biomarker of senescent cells in culture and in vivo. Nat Protoc 4(12): 1798-1806. 10.1038/nprot.2009.191 20010931

[4] Deruy E., Gosselin K., Vercamer C., Martien S., Bouali F., Slomianny C., Bertout J., Bernard D., Pourtier A. and Abbadie C. (2010). MnSOD upregulation induces autophagic programmed cell death in senescent keratinocytes. PLoS One 5(9): e12712. 10.1371/journal.pone.0012712 PMC2939051 20856861

[5] Deruy E., Nassour J., Martin N., Vercamer C., Malaquin N., Bertout J., Chelli F., Pourtier A., Pluquet O. and Abbadie C. (2014). Level of macroautophagy drives senescent keratinocytes into cell death or neoplastic evasion. Cell Death Dis 5(12): e1577. 10.1038/cddis.2014.533 PMC4649843 25522271

[6] Di Micco R., Krizhanovsky V., Baker D. and d'Adda di Fagagna F. (2021). Cellular senescence in ageing: from mechanisms to therapeutic opportunities. Nat Rev Mol Cell Biol 22(2): 75-95. 10.1038/s41580-020-00314-w PMC8344376 33328614

[7] Evangelou K., Lougiakis N., Rizou S. V., Kotsinas A., Kletsas D., Munoz-Espin D., Kastrinakis N. G., Pouli N., Marakos P., Townsend P., .(2017). Robust, universal biomarker assay to detect senescent cells in biological specimens. Aging Cell 16(1): 192-197. 10.1111/acel.12545 PMC5242262 28165661

[8] Gosselin K., Deruy E., Martien S., Vercamer C., Bouali F., Dujardin T., Slomianny C., Houel-Renault L., Chelli F., De Launoit Y., .(2009). Senescent keratinocytes die by autophagic programmed cell death. Am J Pathol 174(2): 423-435. 10.2353/ajpath.2009.080332 PMC2630552 19147823

[9] Gosselin, K., Martien, S., Pourtier, A., Vercamer, C., Ostoich, P., Morat, L., Sabatier, L., Duprez, L., T'Kint de Roodenbeke, C., Gilson, E., et al.(2009). Senescence-associated oxidative DNA damage promotes the generation of neoplastic cells. Cancer Res 69(20): 7917-7925. 10.1158/0008-5472.CAN-08-2510 19826058

[10] Goy E., Tomezak M., Facchin C., Martin N., Bouchaert E., Benoit J., de Schutter C., Nassour J., Saas L., Drullion C., .(2022). The out-of-field dose in radiation therapy induces delayed tumorigenesis by senescence evasion. Elife 11: e67190. 10.7554/eLife.67190 PMC8933005 35302491

[11] Hernandez-Segura A., Nehme J. and Demaria M. (2018). Hallmarks of Cellular Senescence. Trends Cell Biol 28(6): 436-453. 10.1016/j.tcb.2018.02.001 29477613

[12] Li T. and Chen Z. J. (2018). The cGAS-cGAMP-STING pathway connects DNA damage to inflammation, senescence, and cancer. J Exp Med 215(5): 1287-1299. 10.1084/jem.20180139 PMC5940270 29622565

[13] Malaquin N., Martinez A. and Rodier F. (2016). Keeping the senescence secretome under control: Molecular reins on the senescence-associated secretory phenotype. Exp Gerontol 82: 39-49. 10.1016/j.exger.2016.05.010 27235851

[14] Nassour J., Martien S., Martin N., Deruy E., Tomellini E., Malaquin N., Bouali F., Sabatier L., Wernert N., Pinte S., .(2016). Defective DNA single-strand break repair is responsible for senescence and neoplastic escape of epithelial cells. Nat Commun 7: 10399. 10.1038/ncomms10399 PMC4740115 26822533

[15] Tripathi U., Misra A., Tchkonia T. and Kirkland J. L. (2021). Impact of Senescent Cell Subtypes on Tissue Dysfunction and Repair: Importance and Research Questions. Mech Ageing Dev 198: 111548. 10.1016/j.mad.2021.111548 PMC8373827 34352325

[16] Young A. R. J., Cassidy L. D. and Narita M. (2021). Autophagy and senescence, converging roles in pathophysiology as seen through mouse models. Adv Cancer Res 150: 113-145. 10.1016/bs.acr.2021.02.001 33858595

